# An Unusual Case of Ludwig’s Angina Following Mandibular Fracture

**DOI:** 10.7759/cureus.19805

**Published:** 2021-11-22

**Authors:** Leyla Ozbek, Yinan Zhu, Benjamin Olley, Thomas Ringrose, Adrian Farrow

**Affiliations:** 1 Head and Neck Surgery, University College Hospital, London, GBR; 2 ENT, University College Hospital, London, GBR; 3 Oral and Maxillofacial Surgery, University College Hospital, London, GBR

**Keywords:** deep neck space abscess, head and neck trauma, deep neck space infection, mandibular fracture, ludwig's angina

## Abstract

The mandible is the most commonly fractured bone in the maxillofacial region following trauma. Severe infections are rare, and so we highlight an unusual presentation of Ludwig’s angina following a late presentation of a mandibular fracture in a 68-year-old gentleman with significant medical co-morbidities. The recovery process was prolonged and involved multi-disciplinary input. This case makes a recommendation for early recognition of mandibular fractures, antibiotic therapy where appropriate, and hypervigilance when caring for patients with systemic illnesses.

## Introduction

The mandible is the most commonly fractured bone in the maxillofacial region following trauma, with interpersonal violence causing most adult mandibular fractures in western countries [1,2]. Complication rates vary from 7% to 29%, with the most frequent being hyposensitivity of the lip and chin region, disocclusion and infections [3]. Although severe infections following trauma are rare due to the timely fashion in which patients are usually treated, cases can be complicated by systemic illness and smoking, which leads to higher rates of adverse outcomes post-operatively [4]. The development of Ludwig’s angina is a rare complication of mandibular fractures, and is characterised by rapidly spreading diffuse cellulitis of the submandibular, sublingual and submental spaces [5]. We describe here one such case, in particular, highlighting the importance of early treatment and the magnitude of compounding factors, such as other medical co-morbidities.

## Case presentation

A 68-year-old gentleman presented to the ED of a district general hospital in London with a history of worsening jaw pain following a fall at home 10 days prior, for which he had not sought any prior medical advice. History was difficult to obtain as the patient was a non-English speaker, however, reported an inability to eat and drink since the injury. Observations were within normal range and the patient was self-ventilating. On examination, he was found to have a bilaterally tender mandible to palpation with significant left-sided mandibular swelling. He was also found to be in diabetic ketoacidosis as a new presentation of diabetes mellitus (DM), which was stabilised with a sliding scale of insulin. 

A CT scan was performed which showed a displaced left mandibular condylar fracture, undisplaced mandibular symphysis and right mandibular condylar fracture. It also revealed a small collection within the floor of the mouth, and significant soft tissue swelling within the left masticator, parotid and parapharyngeal space displacing the airway to the right.

At this stage, a referral was made to our tertiary oral and maxillofacial surgery department due to suspected Ludwig’s angina, and the patient was transferred to our facility. On examination, the patient had notable swelling and tenderness across the left mandible and submandibular area, with some associated tenderness and swelling on the right side, a raised and tender floor of the mouth, with a maximum mouth opening of 4 cm. 

The patient underwent incision and drainage of Ludwig’s angina in the operating theatre, via external bilateral submandibular incisions, revealing a large amount of pus in the submandibular, sublingual, submasseteric and left parapharyngeal spaces on the left side. The mandibular fractures were repaired with plating via an extra-oral symphyseal approach, and six Penrose drains were placed prior to closure. Due to ongoing airway swelling, the patient returned to the ICU intubated where he remained for four days and was extubated prior to step-down to the ward without further need for tracheostomy.

**Figure 1:**
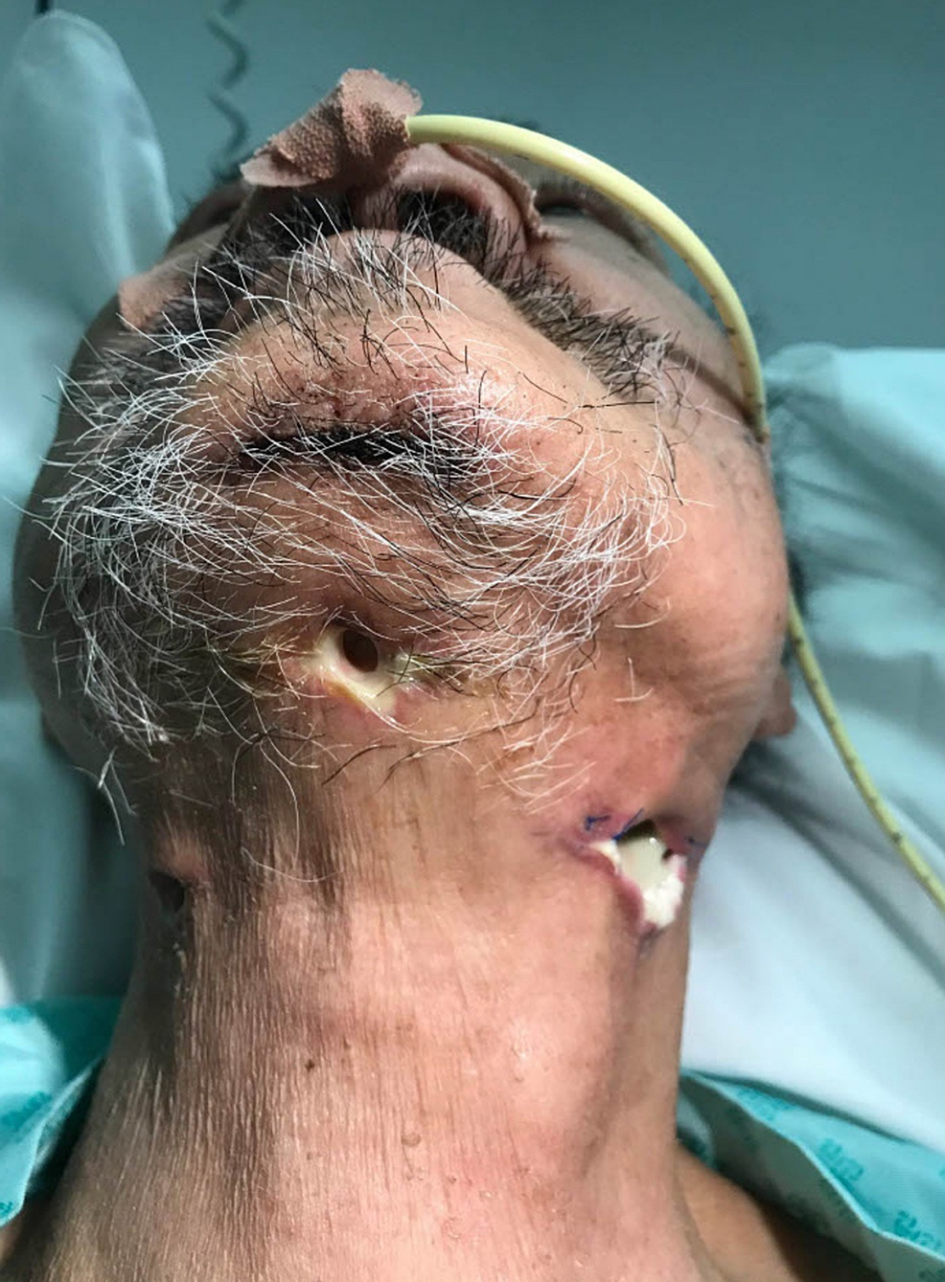
Clinical image showing the non-healing neck wound at 19 days post-operatively.

**Figure 2:**
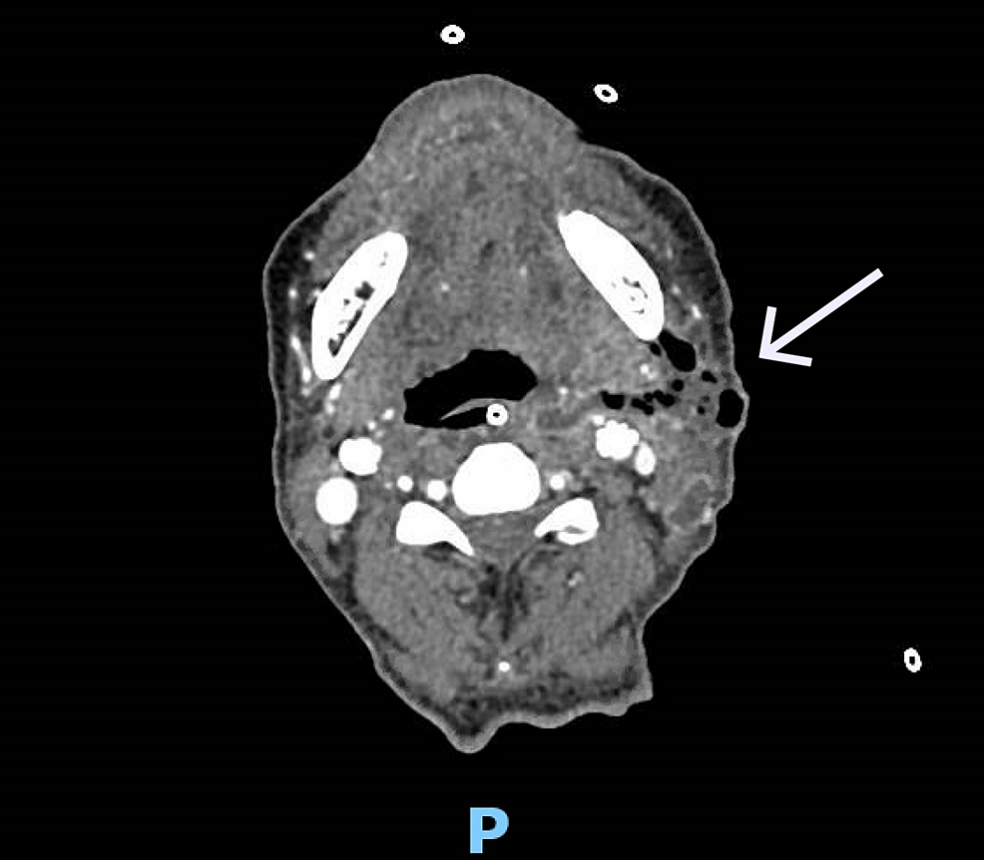
CT neck with contrast 20 days post-operatively showing a complex abscess in the left neck.

**Figure 3:**
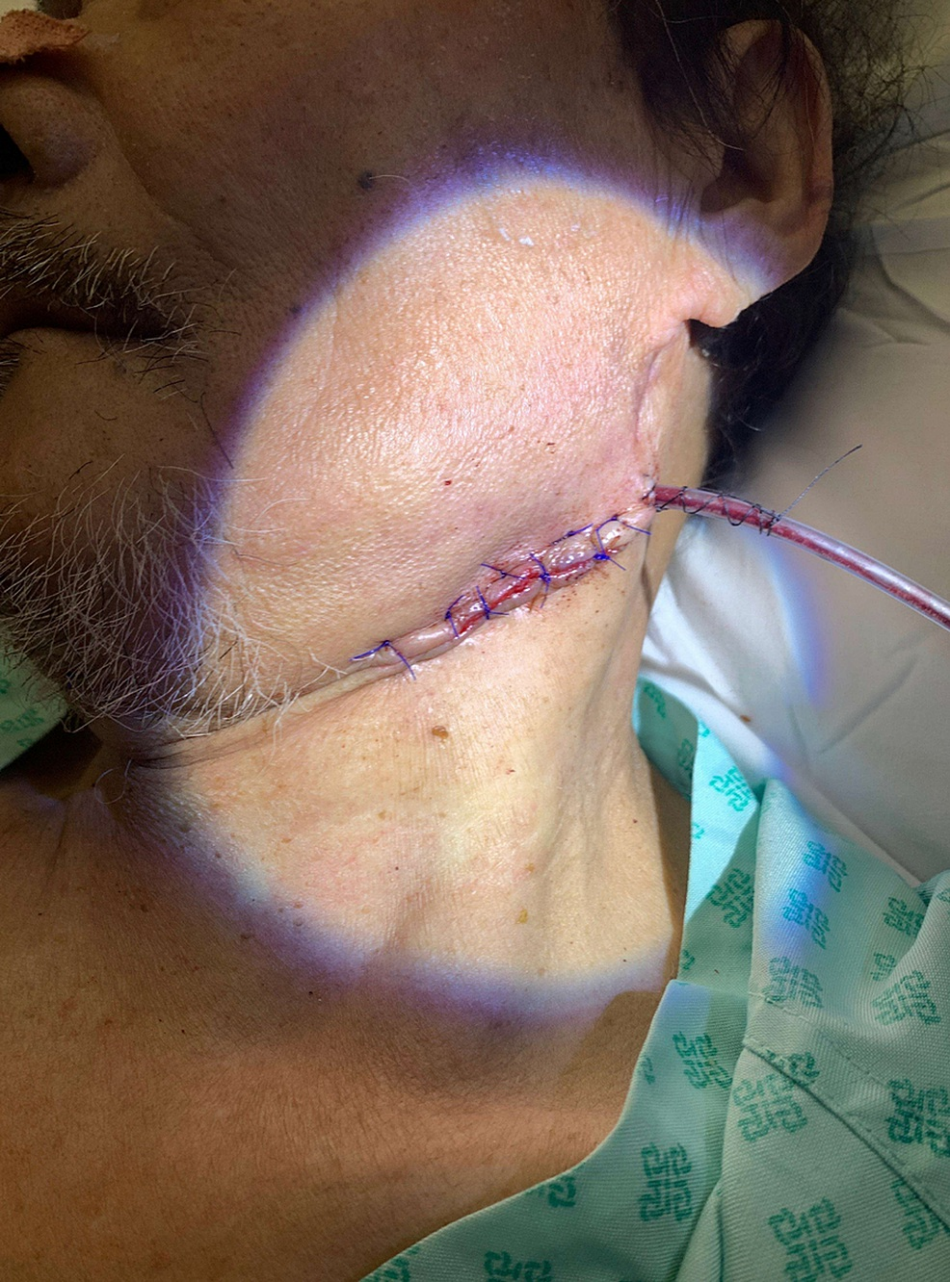
Clinical image showing the wound closed over a Redivac drain at 28 days post-operatively.

Recovery was protracted, and the gentleman remained an inpatient for a further 48 days. There was a significant delay to the healing of the left neck wound, with multiple courses of antibiotics, including piperacillin/tazobactam (Figure 1). CT neck with contrast 20 days post-operatively showed two complex intercommunicating abscesses in the left neck occupying the visceral space and the plane superficial to the sternocleidomastoid that were not amenable to drainage (Figure 2), and the patient received twice-daily washout of the wound in addition to antibiotic therapy. The wound required debriding in the ward and was eventually closed 28 days post-operatively over a Redivac drain (Figure 3). Healing was complicated by a new diagnosis of type one DM and subsequent poor glycaemic control, which rendered the patient immunosuppressed.

## Discussion

The most common cause of Ludwig’s angina is an odontogenic infection, although can also be due to penetrating injury of the floor of the mouth, osteomyelitis or fracture of the jaw, tongue piercing, sialadenitis or sialolithiasis of the submandibular glands [6]. Infection can rapidly progress and can cause a number of potentially life-threatening complications, including airway obstruction, carotid arterial rupture, carotid sheath abscess or aspiration pneumonia [7]. Timely recognition of such patients is therefore crucial, with the cornerstones of management being protection of the airway, surgical drainage of the infection and suitable antibiotic therapy. There are a number of factors causing adverse outcomes in this case, which the clinician must bear in mind when caring for these patients.

Firstly, the delayed presentation allowed the infection to take hold at the site of the mandibular fracture. Maloney P et al. and Anderson T and Alpert B demonstrated that delayed treatment of mandibular fractures showed increased rates of infection [8,9]. This may have been compounded, in this case, by the lack of prophylactic antibiotics, although there is little good quality evidence to support antibiotic prophylaxis for patients with mandibular fractures [10]. There is, however, contradicting evidence to show that delayed fixation of mandibular fractures does not lead to significantly worse outcomes for patients. Hammond D et al. and Lee UK et al. showed that there was no statistical significance in the correlation between time to repair and complication rates, based on a combined study size of 1,213 patients [11,12]. Indeed, the studies by Maloney P et al. and Anderson T and Alpert B were over 25 years ago, and this must be taken into consideration whilst acknowledging that there have been significant advances in trauma management in the interim. Nonetheless, one must bear in mind that the patients in these studies presented to the hospital in a timely fashion following their injury, and spent their time prior to theatre under the care of surgeons on the ward; it can be argued that our gentleman's clinical condition would not have progressed to develop a deep neck space infection was he being monitored in hospital.

Secondly, the importance of the patient’s DM must not be overlooked; the presence of immunosuppressive co-morbidities, most commonly DM, hepatic or haematological disorders have been found to be implicated in the development of severe cervical infections [13], and must therefore be taken into consideration. In particular, DM has been implicated in poor wound healing and prolonged length of inpatient stay due to increased severity of infection [14], which is in accordance with a study by Miller EJ and Dodson TB [15], which showed patients with DM to be more at risk of necrotising fasciitis. Lack of acknowledgement around these issues can cause pitfalls in patient care, and emphasis must be placed on close monitoring, tight glycaemic control and aggressive management of infection [16]. Furthermore, this case brings to light the importance of careful selection of patients for conservative management of mandibular fractures, and that appropriate monitoring frameworks must be in place.

## Conclusions

In conclusion, we highlight the importance of early recognition of mandibular fractures, close monitoring, mitigation of co-morbidities and timely treatment of infection where appropriate, to avoid life-threatening complications such as Ludwig's angina. The absence of such measures can lead to delayed healing and a protracted course of recovery, as was the case with our gentleman, due to his late presentation to the hospital and concurrent new diagnosis of DM which rendered him immunosuppressed. We, therefore, make a recommendation for timely recognition of mandibular fractures, antibiotic therapy where appropriate and hypervigilance when caring for patients with systemic illnesses to minimise adverse outcomes.
